# P-1817. Reducing The Use of Piperacillin/Tazobactam for Common Infections – A Danish Quality Improvement Study

**DOI:** 10.1093/ofid/ofae631.1980

**Published:** 2025-01-29

**Authors:** Trine L Hagen

**Affiliations:** Aalborg University Hospital Thisted, Aalborg SO, Nordjylland, Denmark

## Abstract

**Background:**

During a 4-year period we noticed a doubling of Piperacillin/Tazobactam (PIT) consumption in our hospital, which was not supported by the local antibiotic guideline. This quality improvement study aimed to study prescription behavior. We hypothesized that an intervention might increase guideline adherence, thus decreasing the use of PIT as first line treatment for common infections.

**Methods:**

Baseline data on PIT consumption were collected from April 2022 to March 2023 as defined daily doses (DDD)/100 bed-days. We collected baseline data on guideline adherence from January to March 2023 and performed an audit by reviewing patient charts. A multifaceted intervention was carried out from April 1^st^ to December 31^st^, 2023, consisting of teaching sessions, feedback to prescribers, discouraging use of urinary dipsticks, creating pocket guidelines, and revision of the antibiotic guideline.

Inclusion criteria: Immuno-competent patients ≥ 18 years being prescribed PIT for community-acquired infections. Patients with immunosuppression or at risk of hospital-acquired infection were excluded.

We compared the number of PIT prescriptions between baseline and intervention by using unpaired t-tests. Change in adherence to guidelines was tested using the Chi-squared test.

The primary outcome was reduction in PIT use and secondary outcome was increased guideline adherence.

**Results:**

Three-hundred and sixty-nine patients were included. During the intervention, we observed a 26% decrease in PIT use (DDD/100 bed days) and 41% reduction in the number prescribed PIT doses/week. Correctly prescribed PIT doses increased from 49.6% to 53.8% after the intervention. The correct prescriptions were differentiated between senior (75%), junior (57.7%) and locum doctors (43.5%).

**Conclusion:**

By using a multifaceted intervention strategy to encourage prescribers to follow local guidelines, we observed a substantial reduction in the use of PIT for community-acquired infections in immuno-competent patients. Forming a formal antimicrobial stewardship team may play an important role in maintaining vigilance towards antibiotic prescribing behavior.

**Figure:**
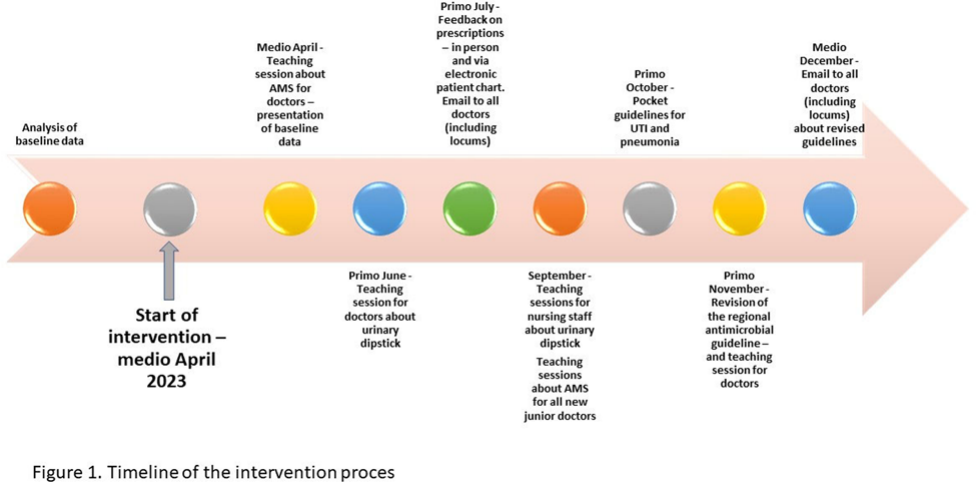

**Figure:**
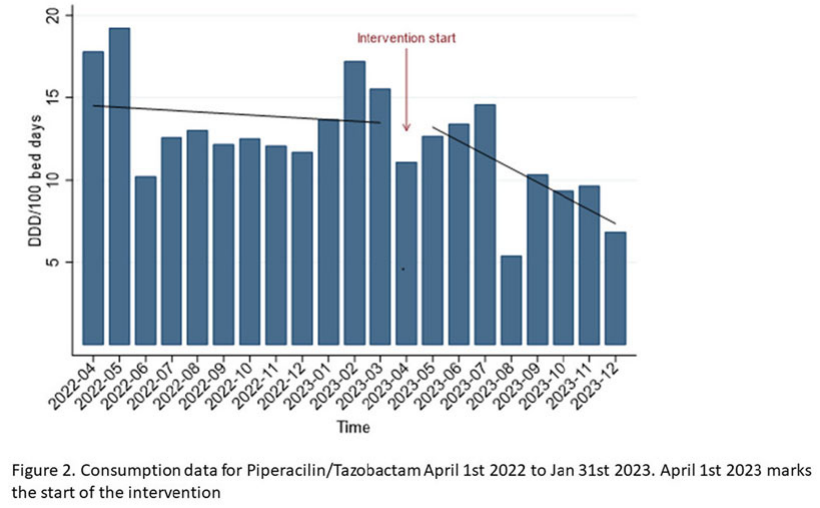

**Figure:**
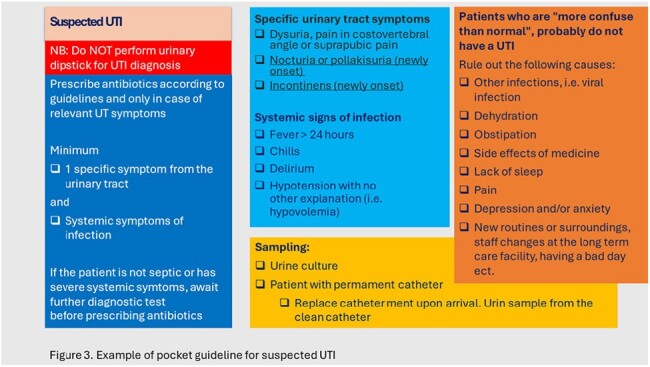

**Disclosures:**

**All Authors**: No reported disclosures

